# Gender relations and health research: a review of current practices

**DOI:** 10.1186/1475-9276-10-60

**Published:** 2011-12-13

**Authors:** Joan L Bottorff, John L Oliffe, Carole A Robinson, Joanne Carey

**Affiliations:** 1 School of Nursing, University of British Columbia's Okanagan Campus, 3333 University Way, Kelowna, BC V1V 1V7, Canada; 2 Centre for Social, Spatial and Economic Justice, University of British Columbia's Okanagan Campus, 3333 University Way, Kelowna, BC V1V 1V7, Canada; 3 School of Nursing, University of British Columbia's Vancouver Campus, 302-6190 Agronomy Road, Vancouver, BC V6T 1Z3, Canada

**Keywords:** gender relations, gender, femininities, masculinities, health, illness experiences

## Abstract

**Introduction:**

The importance of gender in understanding health practices and illness experiences is increasingly recognized, and key to this work is a better understanding of the application of gender relations. The influence of masculinities and femininities, and the interplay *within and between *them manifests within relations and interactions among couples, family members and peers to influence health behaviours and outcomes.

**Methods:**

To explore how conceptualizations of gender relations have been integrated in health research a scoping review of the existing literature was conducted. The key terms *gender relations*, *gender interactions*, *relations gender*, *partner communication, femininities and masculinities *were used to search online databases.

**Results:**

Through analysis of this literature we identified two main ways gender relations were integrated in health research: a) as emergent findings; and b) as a basis for research design. In the latter, gender relations are included in conceptual frameworks, guide data collection and are used to direct data analysis.

**Conclusions:**

Current uses of gender relations are typically positioned within intimate heterosexual couples whereby single narratives (i.e., either men or women) are used to explore the influence and/or impact of intimate partner gender relations on health and illness issues. Recommendations for advancing gender relations and health research are discussed. This research has the potential to reduce gender inequities in health.

## Introduction

Health is affected by macro-level influences including social structures and institutions which shape the expectations of women and men, and the way their lives are organized [1]. To understand health practices and illness experiences it is increasingly recognized that accounting for gender is vital [2,3]. Gender, defined as the socially prescribed and experienced dimensions of femininity and masculinity in society, is evident in the diverse ways individuals engage in health behaviours [2].

In men's health literature, hegemonic masculinity has been associated with risk taking behaviours that compromise health and illness outcomes [4-8]. Conceptualizations of masculinities have also been used to examine an array of issues such as men's depression [9,10], prostate cancer [11] and testicular cancer [12]. Men's diet behaviours and food choices [13-16], tobacco use patterns [17] as well as help-seeking behaviours [18-20] have also been described in relation to masculinities. In contrast to the uptake of masculinities in men's health research, Lyons [14] points to the dearth of research that examines how femininities influence health experiences despite decades of work examining women's health issues. Researchers who have begun to examine femininity in relation to women's health practices have tended to treat femininity as a uniform concept [21,22]. Understanding the diversity of femininities that influence women's health experiences and behaviours is at a nascent stage.

Although there have been promising developments in accounting for gender influences in health research, the concepts of masculinity and femininity for the most part have been delinked despite the social constructionist premise that gender is relational. Further, this research has been predominantly premised on assumptions of associations between femininity and women, and masculinity and men rather than integrating gender structures that suggest a continuum of experience between men and women, and evolving forms of social relations of gender that influence health [23]. While accounting for a range of social determinants including race, social class, and sexual identity has rendered more sophisticated understandings of men's and women's health, health behaviours need to be understood in the context of men's and women's interactions on both personal and institutional levels [6,14]. There is strong evidence that gender relations both within and between men and women strongly influence health outcomes. For example, individuals who are married engage in more healthful behaviours, report healthier psychological and physical well being, and lower mortality rates compared to divorced, separated, widowed, or single individuals [23,24]. Although marriage is associated with improved health for women and men, its beneficial effects seem to be higher for men. Married men live longer than single men, and widowed men's life expectancy is significantly shortened following the loss of their partners [25-27]. In contrast marriage seems to protect women's health by increasing financial stability [25]. However, married women are more vulnerable than men to negative outcomes of dysfunctional relationships including intimate partner violence. Possible influences underpinning these discordant relationships include feminine ideals around nurturing others and linkages between masculinity and men's disregard for self-health. A gender relations approach recognizes the importance of gender dynamics and the circumstances under which they interact to influence health opportunities and constraints. More than a decade after Schofield, Connell, Walker, Wood, and Butland [4] advocated for increased attention to gender relations by signalling some designated pathways for "doing" gender relations and health research, there appears to be limited uptake of gender relations by health researchers.

The arguments supporting the use of gender theory in health research are compelling - the potential for better science, providing the basis for more effective health care and reducing health disparities [28]. Since gender relations is a cornerstone of gender theory, a scoping review of the empirical literature to describe developments in integrating gender relations in health research is needed to take stock of efforts to incorporate gender relations and provide direction for future research.

In what follows we review the theoretical developments that underpin our understanding of gender relations, review published studies to examine the ways in which the concept of gender relations has been integrated into health research, and offer recommendations for how the field might be advanced.

## Theorizing Gender Relations

Feminist scholars have made significant contributions to conceptualizing gender relations as a set of relationships to address critiques of static and binary constructions of gender and to re-establish gender as socially constructed and relational. They have also advanced understandings of the complex diversity within and across genders by incorporating analysis of other social relationships including class, ethnicity and racialization, and their impact at various ages to acknowledge and anchor the context-specific influences that underlie gender dynamics [29,30].

One of the most influential voices in theorizing gender relations has been that of the Australian sociologist Raewyn Connell [31-33]. While Connell first wrote about *hegemonic masculinity *and corresponding *emphasized femininity *in 1987, it was the former concept that garnered most attention, particularly in men's health research. Connell advanced the theory that masculinities and femininities play out at a societal level, and while there are diverse and multiple forms, all are shaped by the structural influences wherein men dominate women. In recognizing the gender hierarchy, hegemonic masculinity was conceptualized as an idealized masculinity that subordinates other masculinities and femininities [31,34].

Although Judith Butler [35] theorizes that heterosexual desire unites masculine and feminine in a binary and hierarchical relationship, others position gender relations as part of recurring patterns embedded in interpersonal relationships, culture, and social structures and organizations that permeate all aspects of everyday life. Connell [32], for example, conceptualizes gender relations as being part of dynamic social life performed through daily interactions and practices, whereby individual actions collectively constitute and re-create prevailing understandings and enactments of masculinities and femininities but not in a uniform way. She describes four interconnected structures of gender relations: *production relations *reflected in sexual divisions of labour; *power relations *evident in the positioning of men as the dominant class in societal discourses and in the exercise of imperial powers; *emotional relations *that include the influence of hegemonic patterns and relationships in a variety of contexts (e.g., households, workplace); and *symbolic representations *of gender in society [33,36]

Howson's [37] work is an important contribution to gender relations, extending Connell's framework by describing categories of masculinities and femininities as emerging in response to hegemonic masculinity. A plurality of masculinities - *complicit, marginalized, sub-ordinate and protest *are proposed to operate in relation to hegemonic masculinity. In addition, Howson proposes three femininities that function in relation to hegemonic masculinity: *emphasised, ambivalent and protest*. This conceptualization of gender relations challenges constructs of masculinity and femininity as binary opposites amid highlighting the diversity within the gender categories, and the relational gender dynamics in society.

In summary, these theoretical frames provide a useful starting place for examining gender relations in health and hold implications for study designs. First, the relational quality of gender occurs in the interface: i) between masculinities/femininities, ii) among masculinities, and iii) among femininities. Second, the relational interactions can occur across, as well as with, the micro- or interpersonal level and the larger macro- or structural level. Third, conceptualizing gender as relational implies an ongoing, interactive dynamism that is subject to change over time. Forth, gender relations vary according to place such that local geo-political conditions are also significant in generating diversity. Gender relations, therefore, can help us move beyond the dyadic binary gender order that has predominated in health research.

## Approaches to Gender Relations and Health Research

We conducted a scoping review of existing health research to explore the ways gender relations have begun to be taken into account to provide a description of current approaches and provide directions for future research. The key terms *gender relations*, *gender interactions*, *relations gender*, and *partner communication *were used to search online databases including CINAHL, PsychINFO, PubMed, and Sociological Abstracts (1999-2009). Two reviewers independently screened 811 abstracts, and identified 95 potentially relevant manuscripts. The full manuscripts were retrieved and reviewed in relation to criteria for inclusion. Manuscripts were included if they were published, peer-reviewed empirical reports (all types of research) where the primary focus was health and explicit references to gender relations were included in the conceptual framework, study design, or findings. We excluded studies of labour markets and other social structures that reflect gender relations in society when the objectives of the research were not explicitly linked to health. Also excluded were articles that used the term "gender relations" but focused on sex differences or sex-roles. Ten empirical papers met the inclusion criteria. In January of 2010, another search of the CINHAL, PUBMED, PsychINFO, and Sociological Abstracts was conducted using combinations of the terms masculinit*, femininit*, couple intervention, gender, gender relations and *health*. This elicited another four articles that met inclusion criteria.

In group meetings, the authors reviewed the manuscripts, and compared and contrasted the approaches used to incorporate the influence of gender relations. Through this analysis we identified two main ways gender relations were integrated in health research: a) as emergent findings; and b) as a basis for research design. In the latter, conceptualizations of gender relations were included in conceptual frameworks, guided data collection and were used to direct data analysis.

### a. Gender Relations as an Emergent Finding

Gender relations was a concept used by some researchers as a way to interpret their data and in these cases gender relations emerged as a key finding. The contribution of these studies in furthering our understanding of gender relations varied. In some studies, gender relations emerged as a broad inductively derived finding rather than a nuanced gendered perspective and was neither informed by, or integrated with, the theoretical literature. While in other studies rich descriptions of the gender dynamics that emerge out of everyday interactions were provided.

For example, de Vera [38] conducted an ethnographic study to explore factors influencing birth spacing among seven rural Filipino couples using interviews conducted separately with husbands and wives, and supplementary data sources. One of the socio-cultural factors identified to influence birth spacing, labelled as "gender relations," describes the lack of communication between husband and wife, and culturally prescribed gender roles for women as wife and mother. Although some new insights were gained in this study, the concept of gender relations was not explored in an in-depth way using available data.

In a second example, Avotri and Walters [39] interviewed 75 Ghanaian women and found that "relationships with men" was a main theme linked to health problems, and integral to the structure of their lives. The findings were richly detailed and focused on three sub-themes: a) gender division of labour characterized by heavy responsibilities, limited control, and lack of access to resources; b) women's insecurity and vulnerability in their relationships with men where partner expectations were high and power or control was very low; and, c) physical and verbal abuse emergent within intimate relationships. The qualitative findings captured coexisting relational dependency and vulnerability leading to health problems for the women, and illustrated how gender relations could be used to explain the women's health issues.

The descriptive study by Avotri and Walters [39], and others like it, in which gender relations emerges as a key concept or finding have the potential to advance our knowledge of gender relations in several ways. First, linking descriptions of everyday social practices with how gender relations is enacted and the dense social context in which they emerge has the potential to enhance our understanding of gender regimes [40], and the processes by which gender influences health. Second, these emergent gender relations findings sensitize researchers to the central role of gender relations in health and the potential advantages of applying theoretical frameworks of gender relations to future study designs.

### b. Gender Relations as a Basis for Research Design

Gender relations have also been explicitly operationalized in health research as a conceptual framework to shape problem formulation, data collection methods and data analysis approaches and tools. Each is described in more detail in the following sections.

#### Gender Relations as a Conceptual Framework

There are examples in the literature where researchers explicitly set out to examine the link between gender relations and health. In these studies, frameworks to conceptualize gender relations were foundational to study design. Most researchers drew on empirical literature to develop their own conceptual frameworks and included gender relations among a number of other factors. For example, Carter [41] was concerned with the influence of community context on household gender relations in rural Guatemala in an exploration of decision making about health matters. Drawing on findings from qualitative studies regarding contextual factors influencing gender relations, the research team developed a conceptual framework for this study. Using this approach, gender relations were conceptualized as social interactions, grounded in power dynamics between men and women in intimate partnerships, and affected by individual characteristics and contextual variables at household and community levels.

Other researchers developed conceptual frameworks drawing on conceptualizations of masculinities and femininities. Evans et al. [42] for example, focused on gendered dimensions of African Nova Scotians' experiences with breast and prostate cancer. The conceptualization of gender relations underpinning this study focused on masculinities, femininities, and the hegemony of idealized masculinity with its implications for sex-specific cancer care. In a similar way, Landstedt, Asplund, and Gadin [43] drew on the work of Connell [31,44] and were concerned with masculinities, femininities, power relations, and the reciprocal influence between gender practices and societal structures in positioning their study of adolescent mental health.

It is noteworthy that none of the studies provided a clear definition of gender relations as part of the conceptual framework underpinning this research. Nevertheless, such efforts to integrate the concept of gender relations within conceptual frameworks have served, in part, to foreground gender relations in health research. In contrast to these approaches, there are a few studies that have made explicit use of gender relations theory to anchor their research [45-47]. In each of these studies, Howson's schema was used to advance gender relations as a conceptual foundation and the pathways reflected in Howson's work were used to purposefully guide methodological approaches to data collection and analyses as well as the discussion of the findings.

#### Gender Relations: Developments in Data Collection

The integration of gender relations in health research has prompted important developments in quantitative and qualitative data collection methods to enhance the potential for examining the relationship between gender as a social dynamic and health.

Quantitative researchers have used a variety of approaches to measure gender relations including combinations of commonly used socio-structural variables. In an examination of gender relations at a society level, Chun, Khang, Kim, and Cho [48] hypothesized that gender inequities in Korean society might explain women's high morbidity, despite increasing prosperity in the country as a whole. The influence of gender relations was measured indirectly using existing survey data related to socio-structural determinants and included marital status, living arrangements, education, occupation, and employment status. Marital status, for example, was conceptualized as an important socio-structural factor that negatively influenced women's health in a patriarchal culture pointing to the obligations associated with women's gendered roles and the "double burden" of working, married women [48]. Others have used measures specifically designed to assess dimensions of gender relations. For example, Hunt [49] used the BEM Sex Role Inventory to examine "gender-related" experiences and health among two cohorts of women. Carter [41], on the other hand, designed four questions to "measure directly some aspects of gender relations and husbands' authority" in the aforementioned study of Guatemalan women. The questions focused on who keeps (guards) money for household expenses, who decides which health care provider to see when sick, what medicine is purchased, and what food to buy.

Other health researchers have used qualitative data collection approaches to examine the influence of gender relations. Semi-structured individual interviews have been used by some researchers. Bottorff et al. [50] used Howson's [37] framework of gender relations as a conceptual lens for examining heterosexual couples' tobacco use patterns. Adopting parallel semi-structured interviews with women and their male partners, the researchers asked participants to describe their interactions with their partners, and whether these interactions undermined or promoted tobacco reduction. Interviewers encouraged participants to provide examples of what might be overheard by someone listening to their conversations with partners related to smoking. Individual interview data with male and female partners were then brought together using dyad summaries to construct couple-level data related to interaction patterns and to facilitate an analysis of gender relations and comparisons within and between couple dyads [49,50].

Focus groups have also been used as a means to better understand gender relations in the context of norms related to sexual practices and HIV protection in several African locations [24,25,51]. These studies involved men and women in same-sex focus group interviews using similar questions to facilitate data comparisons. Ndinda et al. [24] were explicit about how they framed focus group questions to explore gender relations (e.g., Who generally decides on the use of contraception, condom use and child bearing in a sexual relationship? Can a woman say no to sex?). In Tolhurst et al.'s [26] investigation of how "gendered dynamics" within intra-household bargaining influenced seeking health care for children in the Upper Volta region of Ghana, focus group data were supplemented with a variety of qualitative and participatory methods including role-plays, pile-sorting exercises, community mapping, and wealth/wellbeing ranking exercises, key informant interviews, in-depth individual interviews and critical incident interviews.

Evans et al. [42] made use of both mixed and single-sex focus groups to describe the influence of gendered and cultured relations on experiences of breast and prostate cancer among Africans living in Nova Scotia, Canada. In this study, focus group questions directly addressed gender relations: "What is the role of men and women in your community? What does being masculine and feminine mean to you? How has cancer affected how your body works/looks? How has cancer affected your relationship with your partner, family, friends, and community?"(p. 262) [42].

These studies illustrate that approaches to gender relations data collection are diverse and emergent. Efforts to include the voices of *both *men *and *women in studies of heterosexual gender relations are evident and point the way for exploration of other forms of gendered relations. There is also a need to develop measures of gender relations, and the current reliance on qualitative approaches, while reflecting the early stage of development in the field, might also garner gender relations items for inclusion on survey questionnaires.

#### Gender Relations as an Analytical Tool

Qualitative researchers have made explicit use of conceptualizations of gender relations as analytical tools. We describe three studies to highlight this methodological approach.

Bottorff et al. [47] interviewed women about the smoking practices of their men partners in the context of pregnancy and the postpartum. Howson's [37] framework was used as an analytical tool for questioning and interpreting women's narratives to examine how they constructed men's behaviors in relation to smoking and masculinity, and the way that they positioned their efforts to influence men's smoking.

Another study drawing on Howson's [37] and Schippers [27] theorizing in gender relations focused on how masculinities and femininities were operationalized among heterosexual couples in relation to food and diet in the context of prostate cancer [45]. Individual semi-structured interviews with men and their women partners were analyzed to identify and understand how gender relations in heterosexual couples influenced men's diets.

Additional advantages of using a gender relations approach are reflected in a study by Oliffe et al. [46] that examined men's depression through interviews with men who were formally diagnosed and/or self-identified as depressed, and their female partners. In this study each couple was assigned a particular gender relations category inductively derived from an analysis of the way depression-related couple interactions played out. For example, "trading places," embodied by most couples, was a pattern in which men were prepared to stay at home and assume domestic responsibilities while women took on 'breadwinning' responsibilities. Such an arrangement permitted men to manage their depression at home, avoid seeking professional help, and conceal the losses and deficits that depression posed for their masculinity. The study, drawing on Howson's [37] framework, concluded that examining hegemonic femininity (the feminine aspects of idealized heterosexual relationships) as well as pariah femininities (hegemonic masculine characteristics or practices that, when embodied by women, are simultaneously stigmatized and feminized), and male femininities was well founded [27].

## Discussion

Despite the growing attention to gender relations by theorists, the relatively small body of empirical health research that explicitly and purposefully incorporates gender relations suggests that this field of research is at a beginning stage. When researchers acknowledge the importance of gender relations to health practices and experiences, the degree to which they define and engage with gender relations varies considerably. In addition, it is noteworthy that the studies included in this review were predominantly focused on health behaviours and on interpersonal interactions rather than the influence of meso/macro level representations of gender relations on health. Although it is possible that other population-based gender studies were not identified, this may be a reflection of the nascent stage of the research. The lack of consistent language regarding gender relations may have also limited the number of papers included in this review. We often found that while the term 'gender relations' was used, gender relations was not directly addressed in the research. Some authors used sex and gender interchangeably, and while some defined *gender*, the concept of gender relations was rarely clearly articulated. Nevertheless, this review provides useful insights into this emerging area of research and points to key areas where developments are needed.

Conceptual clarity in the use of gender relations is clearly needed to strengthen health research. Recent efforts to use gender relations theoretical frameworks as a conceptual basis for research and to guide data collection and analysis are promising and afford momentum and direction for advancing the field. However, the concept of femininities in health research needs more attention along with broader considerations about what constitutes ideals in the context of gender relations between and among men and women. In this way, perpetuating the binary conceptualization that positions men and women as opposites might be avoided by paying attention to what, as well as how, specific relations work for and against health and well-being.

Although gender relations has featured most prominently in ethnographic work dedicated to understanding health practices in developing countries, the potential for studying men's and women's health behaviour in western societies and in micro yet increasingly globalized contexts is ever present. Wherever this research is conducted, gender relations and health studies will be strengthened by ensuring that diverse perspectives are included. The study of interactional patterns between and among women and men does not adequately distil gender relations unless a gendered perspective is taken. For example, in studies of gender relations in households the perspective of both partners is required, and research must extend beyond heterosexual couples to include same-sex relations and other types of family and peer relations. Although the identification and use of standard indicators for gender relations would allow researchers to account for gender relations (e.g., as a confounder or independent variable) in survey research, the complex, social terrain in which gender relations emerge is likely to require multi-dimensional measures developed for application to specific societal and cultural contexts.

The integration of gender relations in health research will also be advanced through sharing the details about how this work is and can be done. We often found that descriptions of data collection methods aimed specifically at capturing gender relations were missing or limited to a sentence or two. Difficult to determine, for example, in qualitative studies was how gender relations were captured through specific interview questions or observations. Methodological challenges reside here, and need to be acknowledged and addressed. If we take direction from contemporary theorists that gender relations are multiple and have components of hegemony and power dynamics, recognizing when these dynamics influence data collection is also important to modifying approaches to ensure the safety of vulnerable participants (e.g., partner conflict and/or abuse). Although conjoint interviews provide an opportunity to observe gender relations, there may be situations where these interviews place individuals at risk [52].

Theorists have identified locations or settings where gender relations might be best studied. For instance, gender as relational experience occurs on personal and intimate levels as well as on cultural and institutional levels [4,6,53]. This suggests that gender relations and health studies can and should occur in diverse locations and contexts to more fully apprehend the multiplicity and patterns within productions of gender relations and their influence on health.

## Conclusions

Gender relations are an exciting and emergent area in need of more attention from health researchers. Health-related behaviours do not operate in isolation and need to be understood in the context of interactions within and between men and women across personal, interpersonal and institutional levels. A better understanding of gender relations and health in research and policy will have direct implications for health interventions and guide decisions about whether group, dyadic or single point programs are likely to be effective. In addition, this research has great potential to challenge relational patterns that are so often taken-for-granted and contribute to reducing gender inequalities in health.

## Competing interests

The authors declare that they have no competing interests.

## Authors' contributions

JB lead this project, participated in the study design and conduct of the research, and contributed to preparation of the manuscript. JO participated in study design and data analysis, and contributed to preparation of the manuscript. CR participated in study design and data analysis, and contributed to preparation of the manuscript. JC performed the literature searches and data extraction, and assisted with preparation of the manuscript. All authors read and approved the final manuscript.
